# Orthogonality in Principal Component Analysis Allows the Discovery of Lipids in the Jejunum That Are Independent of Ad Libitum Feeding

**DOI:** 10.3390/metabo12090866

**Published:** 2022-09-14

**Authors:** David Balgoma, Fredrik Kullenberg, Karsten Peters, David Dahlgren, Femke Heindryckx, Hans Lennernäs, Mikael Hedeland

**Affiliations:** 1 Analytical Pharmaceutical Chemistry, Department of Medicinal Chemistry, Uppsala University, 751 23 Uppsala, Sweden; 2 Unidad de Excelencia, Instituto de Biología y Genética Molecular (IBGM), Universidad de Valladolid—Consejo Superior de Investigaciones Científicas (CSIC), 47003 Valladolid, Spain; 3 Translational Drug Development and Discovery, Department of Pharmaceutical Biosciences, Uppsala University, 751 23 Uppsala, Sweden; 4 Department of Medical Cell Biology, Uppsala University, 751 23 Uppsala, Sweden

**Keywords:** anthracyclines, unsupervised analysis, lipidomics, lipidome

## Abstract

Ad libitum feeding of experimental animals is preferred because of medical relevance together with technical and practical considerations. In addition, ethical committees may require ad libitum feeding. However, feeding affects the metabolism so ad libitum feeding may mask the effects of drugs on tissues directly involved in the digestion process (e.g., jejunum and liver). Despite this effect, principal component analysis has the potential of identifying metabolic traits that are statistically independent (orthogonal) to ad libitum feeding. Consequently, we used principal component analysis to discover the metabolic effects of doxorubicin independent of ad libitum feeding. First, we analyzed the lipidome of the jejunum and the liver of rats treated with vehicle or doxorubicin. Subsequently, we performed principal component analysis. We could identify a principal component associated to the hydrolysis of lipids during digestion and a group of lipids that were orthogonal. These lipids in the jejunum increased with the treatment time and presented a polyunsaturated fatty acid as common structural trait. This characteristic suggests that doxorubicin increases polyunsaturated fatty acids. This behavior agrees with our previous in vitro results and suggests that doxorubicin sensitized the jejunum to ferroptosis, which may partially explain the toxicity of doxorubicin in the intestines.

## 1. Introduction

**Figure 1:**
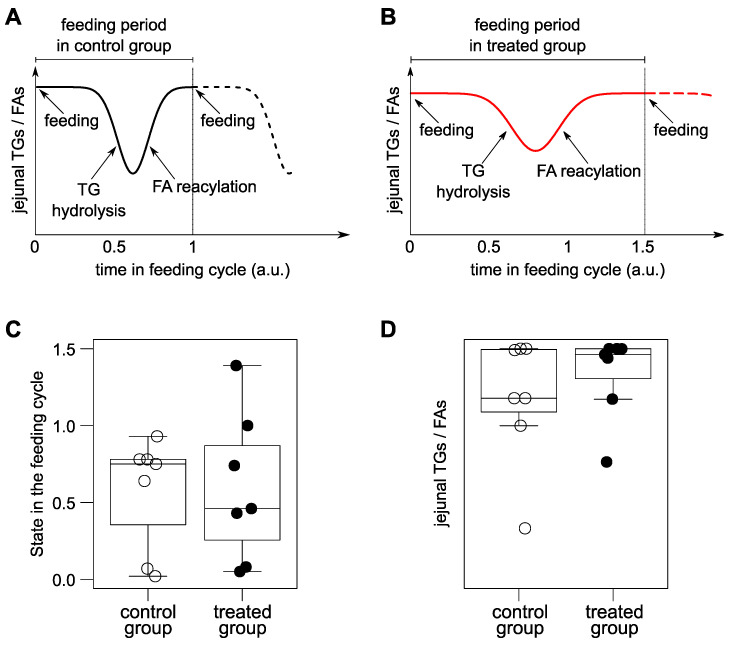
Simulation of the effect of ad libitum feeding on the ratio TGs/FAs in the jejunum. (**A**) Illustration of the change of the ratio TGs/FAs in the jejunum during the feeding period. (**B**) Illustration of the change of the feeding period in a treatment that increases the feeding period by 50% and affects the length of stay of the bolus in the jejunum. (**C**) Simulation of ad libitum feeding on the point of the animals in the feeding cycle. The position of the animals in the feeding cycle at sacrifice was simulated by sampling a uniform distribution between 0 and 1 for the control group (n = 7) and sampling a uniform distribution between 0 and 1.5 for the treated group (n = 7). (**D**) Values of the ratio TGs/FAs of the simulations in panel C, according to the curves shown in panels A and B. Ad libitum feeding provokes that the ratio TGs/FAs is not matched between the two groups. In addition, even if it would be paired by chance, the same ratio TGs/FAs could correspond to two different points in the digestion: when TG hydrolysis dominates or when FA acylation dominates. Consequently, even if the average ratio TGs/FAs is matched, the animals could be in two different digestion states and bias the analysis.

Rats (and mice) are the most common pre-clinical models to study the effect of different treatments, including the effect on the metabolism of lipids. As feeding temporarily affects the lipidome, rats should ideally fast before sacrifice. However, because of their fast metabolism, rats stress and perform coprophagy. Consequently, ethical committees in many countries require ad libitum feeding of the animals. This request is stronger in animals that are already exposed to stress or suffering (such as administration of chemotherapeutic drugs). This requirement limits the validity and relevance of these pre-clinical models to investigate the metabolism of lipids in detail. In addition, one expects that the interference of ad libitum feeding to be especially strong in the tissues involved in digestion, such as the intestine and the liver. During ad libitum feeding, intestinal tissues isolated from different animals can be at different stages of digestion, depending on the timing of their last feeding before sacrifice (Figure 1). In addition to the random point in the digestion cycle at sacrifice, the duration of the feeding cycle and the amount eaten may change with the treatment, inducing an extra interference in matching the control and the treated groups (Figure 1).

Regarding lipids, digestion is characterized by the hydrolysis of triacylglycerols (TGs) [1]. First, TGs are hydrolyzed to a 2-monoacylglycerols (2-MGs) and free fatty acids (FAs). Subsequently, FAs are reacylated to the 2-MGs so TGs are reconstructed to build intestinal lipoproteins (mainly chylomicrons) in the enterocytes [1]. In this process, the regiosomeric composition of TGs is of special interest, as it affects the absorption of different FAs [1,2]. Consequently, regarding the jejunum, one expects that the ratio jejunal-TGs/jejunal-FAs is lower when the isolated jejunum is in digesting state than when the animal has not eaten recently (Figure 1A,B). In addition, one expects that the digestion of other lipids [3] and the increase of FAs during digestion also effects the rest of the lipidome in the jejunum (specially glycerophospholipids). Consequently, the ethical and technical requirement of ad libitum feeding of rats is expected to scramble the effects of any studied treatment on the jejunal lipidome. In addition, ad libitum feeding is expected to scramble the liver lipidome. This is because digested lipids in both lumen and enterocytes are directly transported to the liver through the portal vein, and indirectly via lymphatic drainage in the central circulation [1].

In addition to the random effect, many treatments affect the frequency of feeding, which makes it difficult to match control and treated groups (Figure 1). Consequently, regarding the hepatic or jejunal lipidomes of animals fed ad libitum, when supervised pre-clinical models are applied (e.g., orthogonal partial least squares -OPLS-), it is highly plausible that the data analysis captures a putative average effect of the treatments on feeding. In addition, even if the groups feed equally in frequency and amount, it is also plausible that the supervised analysis, by chance, identifies features that seem to be associated with the treatment, but in fact are scrambled by the random feeding (Figure 1). Consequently, the combination of uncontrolled feeding with supervised analysis of the lipidome (and the metabolome) is highly probable to yield irreproducible false discoveries.

In contrast to supervised analysis, orthogonality in unsupervised principal component analysis may identify metabolic traits in the jejunum and the liver that are statistically independent of the digestion status of the tissue. This is possible because principal component analysis summarizes the data by reducing their dimensionality into new variables (principal components) that: (i) are ordered by their variation, and (ii) are statistically independent (orthogonal) [4,5,6]. If one analyzes the jejunum in the context of uncontrolled digestion status (Figure 1), one expects that ad libitum feeding will induce a high variability in the lipidome. Specifically, one expects that FAs and TGs in the jejunum anticorrelate because of the process of luminal hydrolysis and intracellular reacylation during digestion. Consequently, in a principal component analysis of the jejunum lipidome, one expects that the main principal component is dominated by FAs and TGs, having FAs and TGs opposite sign in their loadings. This principal component would be associated to the digestion status of the isolated jejunum. From a statistical point of view, a lipid or metabolite that presents low absolute loading in this “digestion” principal component but high absolute value in another principal component may contain biological information that is statistically independent of the confounding of ad libitum feeding. Consequently, these orthogonal metabolites would be associated to the treatment, not to the uncontrolled feeding.

In addition to the orthogonality to the digestion principal component, the lipids selected should be pathway consistent. By this expression, we mean that similar lipids that are synthesized by the same pathway should present similar behavior in the principal components. Consequently, they should yield similar loadings in the principal components (i.e., cluster in a close area when the loadings of the principal components are represented).

In this context, we aimed to explore the possibility of isolating lipids that were statistically independent of the digestion status of the animal at sacrifice. To achieve this aim, we treated rats with doxorubicin (DOX), a chemotherapeutic drug known to influence tissue metabolism, while they were fed ad libitum. Then, we analyzed the lipidome of the jejunum and the liver of the rats by mass spectrometry coupled to liquid chromatography. Subsequently, we performed principal component analysis of the jejunum lipidome and studied the association between the components and the variation of FAs and TGs. Finally, we combined the data from the jejunum and the liver to investigate the possibility of isolating hepatic lipidomics traits statistically independent of the ad libitum feeding. With this strategy, we could identify a group of lipids with polyunsaturated fatty acids (PUFAs) in the jejunum that: (1) were statistically independent of the ad libitum feeding; (2) were pathway consistent; and (3) increased along time in the jejunum of animals treated with DOX.

## 2. Results and Discussion

### 2.1. Principal Component Analysis Allowed the Identification of Lipids That Were Statistically Independent of Ad Libitum Feeding in the Jejunum (but Not in the Liver)

**Figure 2:**
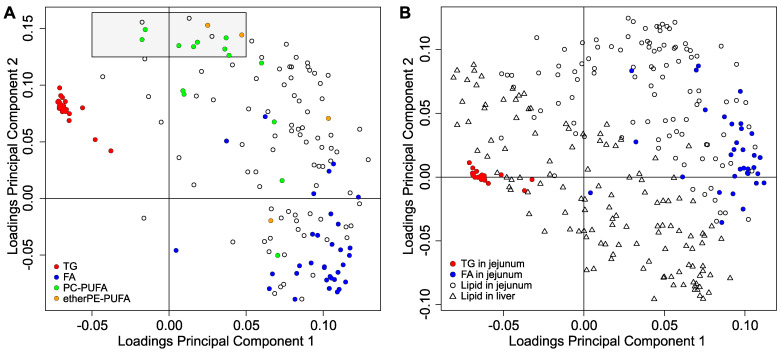
Loadings of the principal component analysis of the jejunal and hepatic lipidomes. (**A**) Loadings plot of the first and second principal components of the lipidome in the jejunum. Triacylglycerols (TGs) in red; free fatty acids (FAs) in blue; polyunsaturated−fatty−acid−containing phosphatidylcholines (PC−PUFA) in green; and polyunsaturated−fatty−acid−containing etherglycerophospholipids of ethanolamine (etherPEs−PUFA) in orange. (**B**) Loadings plot of the first and second principal components of the principal component analysis of joining the lipidomes of the jejunum (circles) and the liver (triangles). Jejunal TGs in red and jejunal FAs in blue. The scores of both models can be found in Figure S1.

To study the possibility of extracting lipids in the jejunum that were statistically independent of the variation induced by the ad libitum feeding, we analyzed the lipidome of the jejunum by principal component analysis. To achieve this aim, we represented the loadings of the first and the second components (Figure 2A).

The first principal component in the jejunum (abscissa in Figure 2A) was dominated by FAs (blue points) and TGs (red points). FAs and TGs presented the opposite sign of the loadings. From a statistical point of view, this means that they anticorrelate, i.e., when TGs are high in a relative way, FAs are low in a relative way (and vice versa). As reasoned before, this variation is the expected statistical manifestation of isolating the jejunum of different animals at different digestion status, i.e., ad libitum feeding. Consequently, we associated the first principal component of the lipidome in the jejunum to the digestion status of the jejunum. This variation represented 32% of the variance of the principal components and was the main effect on the lipidome of the jejunum. Consequently, in our study, the main source of variation was the digestion status of the jejunum (and consequently the first principal component is associated to the digestion status by ad libitum feeding). Nevertheless, in other studies, the effect of the treatment on the variation of the dataset might overcome the variation induced by ad libitum feeding. In that case, the principal component associated with the digestion status might be the second or higher order principal component.

Despite digestion dominated the lipidome of the jejunum (Figure 2A), a group of lipids presented: (i) high positive loadings for the second principal component (ordinate axis in Figure 2A), and (ii) low absolute loadings for the first principal component (highlighted with a gray rectangle in Figure 2A). From a statistical point of view, they are orthogonal to the first principal component. By the properties of the principal component analysis, this means that, in our dataset, they were statistically independent of the digestion status in the jejunum (first component). These lipids belonged to two families: (I) PUFA−containing etherglycerophospholipids of ethanolamine (etherPE−PUFA); and (II) PUFA−containing glycerophospholipids of choline (PC−PUFA). It could be argued that the statistical orthogonality of a lipid to the first principal component (digestion dominated) could happen by chance. However, the selected lipids were pathway consistent. They belonged to specific families and presented PUFAs. Finally, one should note that other lipids may be affected by DOX treatment, as they present loadings with significant absolute value in the second principal component (Figure 2A). Nevertheless, the observed changes of these variables are dominated by ad libitum feeding and scrambled. Consequently, we discarded them from further analysis.

To identify hepatic lipids statistically independent of the ad libitum feeding, we applied principal component analysis to the combination of the data from the liver and the jejunum (Figure 2B). Again, the contrast between jejunal TGs and jejunal FAs (red and blue dots in Figure 2B) dominated the first principal component. Consequently, in the combined analysis of the lipidomes of the jejunum and the liver, the uncontrolled digestion status at the moment of sacrifice of the animals was the main source of variation. In addition, the lipids in the liver were spread along the first principal component (triangles in Figure 2B). This suggests that changes in the lipids of the liver were dominated by the uncontrolled digestion status of the animal when the tissues were isolated. This influence may be the statistical manifestation of the direct association of the intestine and the liver by the portal venous system for the transport of absorbed and available lipids [1]. Furthermore, we did not observe hepatic lipids in the liver that were clearly orthogonal to the first component, as we found in the jejunum. Consequently, the analysis in Figure 2B suggests that no lipid in the liver was independent of the ad libitum feeding.

In addition, all families of hepatic lipids were biochemically inconsistent (Figure 2B). For example, PC(18:0/20:4) in the liver was in the second quadrant of the loadings plot (negative first component, positive second component). In contrast, PC(18:1/20:4) (another PC with the same PUFA) was in the fourth quadrant (positive first component and negative second component). This disposition also suggests that the lipidome in the liver was pathway inconsistent because it was scrambled by the uncontrolled feeding of the animals.

**Figure 3:**
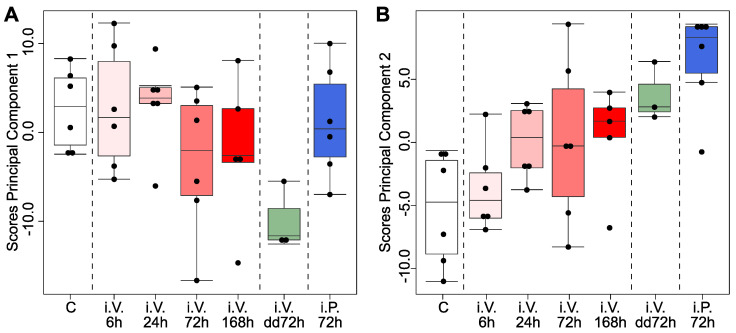
Scores of the principal component analysis of the lipidome in the jejunum. (**A**) Boxplots of the scores of the first principal component of the jejunal lipidome according to the treatment of the animals. (**B**) Boxplots of the scores of the second principal component of the jejunal lipidome according to the treatment of the animals. dd, double dose; i.V., intra venous treatment; i.P., intra peritoneal treatment.

Subsequently, we studied the change of the scores of the first two principal components with the type of treatments (Figure 3). The scores of the jejunal first principal component did not present a clear trend with the treatments (Figure 3A). In contrast, the boxplots of the scores of the second jejunal principal component against the treatments (Figure 3B) presented: (1) a gradual change with the time of the treatment in relation to the control group [increase from 24 h after intra venous treatment (i.V.) until 168 h]; and (2) a higher score for the strongest or direct treatments in the jejunum [i.V. 168 h; i.V. dd72 h, and i.P. 72 h (intra peritoneal)]. This behavior strongly suggests pathway and treatment consistency for the subgroup of lipids highlighted in Figure 2A. Consequently, the cluster of lipids with PUFAs in the jejunum had regulatory meaning and did not occur by chance.

Taking all these observations together, as we expected, ad libitum feeding scrambled the lipidomes from the jejunum and the liver. Despite this fact, we could identify a subset of lipids in the jejunum that were statistically independent in our dataset from the ad libitum feeding. Consequently, we studied their regulation with the treatment with DOX and the consistency of our observations with previous studies [7].

### 2.2. Doxorubicin Affects Feeding Biomarkers in the Jejunum and Increases Lipids with PUFAs, Which Is Consistent with Previous Results in Cell Cultures

DOX may affect the lipidome of the jejunum by: (1) affecting the frequency and amount of feeding and (2) regulating the lipids that were statistically independent of the feeding.

To examine the effect on the lipidome by the average feeding frequency, we defined a lipidomics-based fasting score in the jejunum. We defined this score as the logarithm of the ratio sum of TGs/sum of FAs (from the jejunal lipidome). This formula was motivated by the fact that TGs are hydrolyzed into FAs in the lumen and are subsequently transported across the apical enterocyte membrane during digestion.

**Figure 4:**
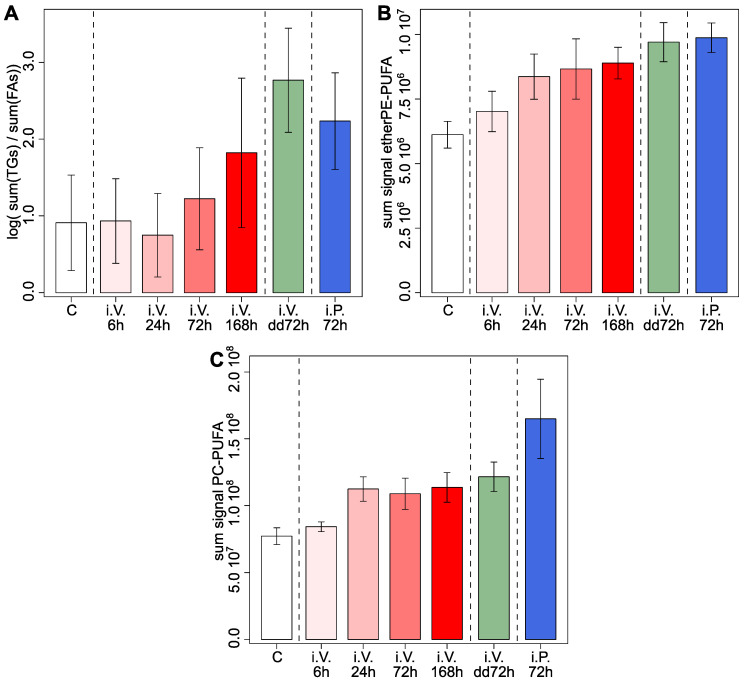
Effect of the treatment in the selected lipids in the jejunum. (**A**) Barplots of the effect of the treatments on the logarithm of the ratio between the sum triacylglycerols (TGs) and the sum of fatty acids (FAs) in the jejunum. (**B**) Barplots of the effect of the treatments on the sum of selected ether glycerophospholipids of ethanolamine with polyunsaturated fatty acids (etherPE−PUFA) in the jejunum. (**C**) Barplots of the effect of the treatments on the sum of selected phosphatidylcholines with polyunsaturated fatty acids (PC−PUFA) in the jejunum. In all cases, the barplots represent the average and the whiskers represent the average plus/minus the standard error of the mean. dd, double dose; i.V., intra venous treatment; i.P., intra peritoneal treatment.

An animal sacrificed during the digestion period will have a lower ratio TGs/FAs in jejunum (Figure 1). Consequently, we expected to have a higher ratio TGs/FAs if a treatment decreases the frequency of feeding. This expected behavior is what we observed in Figure 4A when we represented the lipidomics fasting score. The animals treated with DOX at longer times or the strongest treatments (i.V. 168 h.; i.V. dd72 h, and i.V. 72 h.) presented, on average, a higher lipidomics fasting score. This indicates that the animals with the strongest treatments with DOX presented a lower frequency of feeding. In addition, as control animals eat more frequently than treated animals, this suggests that the scrambling effect of ad libitum feeding was stronger on the lipidome of the control group than on the lipidome of the treated group.

To study the effect of DOX on the lipids with PUFAs, we represented the sum of the signal of these lipids in Figure 4B,C. These two panels show that the sum of etherPEs-PUFA and the sum of PC−PUFAs increased with DOX: (i) in a gradual way with time of exposition (red bars); and (ii) increased strongly with the strongest treatments (green and blue bars). Taken together, this suggests that DOX increased PUFAs in the jejunum. This is in agreement with our previous study, in which we observed a general increase of lipids with PUFAs in in vitro hepatic cell lines treated with anthracyclines [7]. Consequently, despite the confounding factor of ad libitum feeding, this study suggests that anthracyclines also induce PUFAs in the jejunum at in vivo conditions. EtherPE−PUFAs are of special interest, as they have been recently reported to sensitize tumor cells to ferroptosis [8,9].

## 3. Concluding Remarks

Our study confirms that ad libitum feeding scrambles the lipidome of the jejunum and the liver in vivo. Despite this effect, we found that DOX increased two families of lipids with PUFAs in the jejunum (etherPEs and PCs). Four different pieces of evidence support the independent regulation of the digestion status: (i) the loadings of these jejunal lipids were orthogonal (statistically independent) to the principal component dominated by the digestion (Figure 2A); (ii) these lipids were pathway consistent, as they contain PUFAs; (iii) the increase of lipids presented a gradual effect with the strength of the treatment (Figure 3); and (iv) the increase of lipids with PUFAs agrees with our previous results in cell cultures, where the confounding of ad libitum feeding is absent [7]. In conclusion, this combination of facts strongly suggests that DOX increases PUFAs, which play a key role in the sensitivity of cells to ferroptosis [10].

If we had performed supervised OPLS, the supervised analysis would have selected different lipids related to gastrointestinal digestion as the main predictors that would separate the groups. Consequently, it is highly plausible that a supervised analysis would identify average and/or random differences in eating—which is the main effect in the lipidomes according to the principal component analysis in Figure 2. This effect would only disappear if: (i) control and treated animals do not have different feeding habits (frequency and amount), and (ii) all animals are sacrificed at the same time after feeding as circadian rhythm is an important factor. However, as presented in the introduction, there are ethical and technical difficulties to implement these constraints. In our opinion, it is highly plausible that the results from a supervised metabolic analysis of liver or jejunum (and potentially other tissues) from animals fed ad libitum would report non-reproducible discoveries. In addition, because of the contribution of chylomicrons from digestion and very low-density lipoproteins (VLDL) to the blood lipidome, it is highly probable that ad libitum feeding also scrambles the lipidome in blood plasma and serum.

Based on our results, we encourage the scientific community to very carefully interpret the metabolic data from ad libitum fed animals. While we focused on the jejunum and the liver, this is also valid for other organs. As there are ethical and technical limitations for controlling the feeding of rat and mouse pre-clinical models, lipidomics and metabolomics studies should be restricted to specific designs. For example, by pairing healthy and tumor hepatic tissues from the same animal. In this case, one may assume that the ad libitum feeding affects both samples equally and the effect of feeding is matched.

While our pieces of evidence are indirect and focused on a rat model, they agree with the previous knowledge about digestion in mammals (Figure 1). Consequently, it is highly probable that the trials and tribulations of ad libitum feeding reproduce in similar models, such as murine and canine models. Finally, we found specific lipidic traits that were associated to DOX treatment and statistically independent of ad libitum feeding. However, one cannot generalize that finding statistically independent traits is possible with other treatments.

In conclusion, due to the importance of the metabolism in cancer and cancer treatment, a careful analysis of pre-clinical metabolic models with ad libitum feeding is required. Rather than the mechanical application of statistical techniques, such as supervised analysis, context and understanding the limits of statistical analysis are required.

## 4. Materials and Methods

### 4.1. Animal Treatment, Tissue Isolation, and Tissue Homogenization

Doxorubicin (DOX) hydrochloride was purchased from Toronto Research Chemicals, Canada. Stock solutions (100 mg/mL) were prepared by dissolving DOX hydrochloride in dimethylsulfoxide (Sigma-Aldrich, St. Louis, MO, USA) stock solution was then diluted to 5 mg/mL in saline, which was the administered concentration.

Male Wistar Han IGS rats from Charles River Co. (Sulzfeld, Germany and Lyon, France) with body weight 230–470 g and age 6–10 weeks were used. All animals were allowed to acclimatize for at least one week in the Animal Department prior to being used and were allowed water and food ad libitum. Housing conditions were 21–22 °C at a 12–12 h light-dark cycle.

There were seven groups in this study. The control group (n = 6) consisted of intravenous injection of saline and sedated after 6 h. Four groups were treated intravenously with 10 mg/kg of DOX and evaluated in a time-dependent way after 6, 24, 72, and 168 h [respectively, i.V. 6 h (n = 6), i.V. 24 h (n = 6), i.V. 72 h (n = 6), i.V. 168 h (n = 5)]. One group was treated with double dose after 72 h (i.V. 72 h, n = 3). The last group (n = 6) was treated with an intraperitoneal (i.P.) injection of DOX (10 mg/kg) and evaluated after 72 h (i.P. 72 h). Before tissue sampling, the rats were anaesthetized with inactin, jejunal, and liver samples were excised and immediately frozen in liquid nitrogen, and then stored at −80 °C until analysis.

### 4.2. Lipidomics Analysis

The order of sample preparation was randomized. Subsequently, 400 μL of water per 10 mg of tissue were added and homogenized at 4.0 m/s speed using a FastPrep-24 5G homogenizer with Lysing Matrix D (MP Biomedicals, Solon, OH, USA).

To extract the lipids in every sample, 150 µL of homogenate were extracted by Bligh&Dyer method [11]. The chloroformic phases were mixed, evaporated under vacuum, and resuspended in 200 µL of acetonitrile/isopropanol 50:50. A quality control sample was built by pooling 10 µL from every extract. Lipidomics was applied as in Balgoma et al. [12]. Lipids in the extract were separated on an Acquity-UPLC (Waters, Manchester, UK) with a BEH C18 column (1.7 μm, 2.1 × 150 mm) at 55 °C and a gradient of solvents A water/acetonitrile/isopropanol 40:30:30 (*v/v/v*) with 5 mM of ammonium formate, and B acetonitrile/isopropanol 40:60 (*v/v*) with 5 mM of ammonium formate. The gradient (flow 0.275 mL/min) changed linearly from 95% of A at min 0, to 60% at min 3.50, to 37.5% at min 6.00, to 15% at min 9.50, to 1% at min 13.00, which was kept until min 16. The eluent was ionized by electrospray on a Synapt G2S Q-ToF (Waters) scanning between *m/z* 100 and 1200. The injections were randomized, and the quality control sample was injected every seven samples. The samples were analyzed in both positive (2000 V in electrospray ionization) and negative (1500 V in electrospray ionization) modes.

Lipids were identified as described before [7,12], with a limit of absolute deviance of 10 ppm for m/z for the main adducts reported in the literature. For glycerolipids, when more than one fatty acid was possible and the fragmentation signal allowed it, the main combination of fatty acids was determined by the fragmentation patterns, as reported in the literature [13,14]. In addition, for every family of lipids, it is known that the retention time of lipids in C18 chromatography: (i) increases with the number of carbons, and (ii) decreases with the number of unsaturations. These two chromatographic conditions were used to discard false positives. The identification parameters and the signal of the lipids are reported in Supplementary Material 1.

### 4.3. Data Pre-Treatment, Analysis, and Intepretation

For multivariate unsupervised analysis, we used principal component analysis after: (1) log-transformation of the signal of the lipids, (2) centering the variables by the average, and (3) scaling the variables by the standard deviation.

For univariate analysis, we focused on the size of effects, following the recommendations of researchers, statisticians, and the American Statistical Association [15,16,17]. We analyzed the changes in the lipidome by comparing the effect among the treated and the control groups [18]. Consequently, we focused our interpretation in the observed changes in the context of the study, rather than in variables selected by statistical significance [15]. Boxplots are interpreted as: (1) the box represents the first and the third quantiles, (2) the broad horizontal bar represents the median, (3) the upper whisker represents the first quartile plus 1.5 times the interquartile range; and (4) the lower whisker represents the first quartile minus 1.5 times the interquartile range. Barplots are interpreted as: (1) the horizontal line represents the mean; (2) the upper whisker represents the mean plus the standard error of the mean; and (3) the lower whisker represents the mean minus the standard error of the mean.

### 4.4. Software and Graphical Resources

Waters’ Raw data files were transformed into CDF format by Databride (Masslynx 4.1, Waters, Manchester, UK). Mass spectrometric data were pretreated with packages mzR 2.22.0 and XCMS 3.10.2 in R 4.0.3 “Bunny-Wunnies Freak Out”. Principal component analysis was performed by using the function *prcomp* from package *stats* 4.0.3 in R.

Graphical material was generated with R (package *ggplot2* 3.3.2) and processed with Inkscape 1.0.1.

## Data Availability

Data are available in article and Supplementary Material.

## Supplementary Materials

The following supporting information can be downloaded at: https://www.mdpi.com/article/10.3390/metabo12090866/s1, Supplementary Material_1.xlsx: lipid identification and processed data; Supplementary material_2.docx, Supplementary Figure S1.
