# Supplementary material for: Orthogonality in Principal Component Analysis Allows the Discovery of Lipids in the Jejunum That Are Independent of Ad Libitum Feeding
Source: Metabolites. 2022 Sep 14;12(9):866. doi: 10.3390/metabo12090866 (PMC9506031; doi:10.3390/metabo12090866)
Supplement: Supplementary file 1 [file metabolites-12-00866-s001.zip › Supplementary Material_2.pdf]

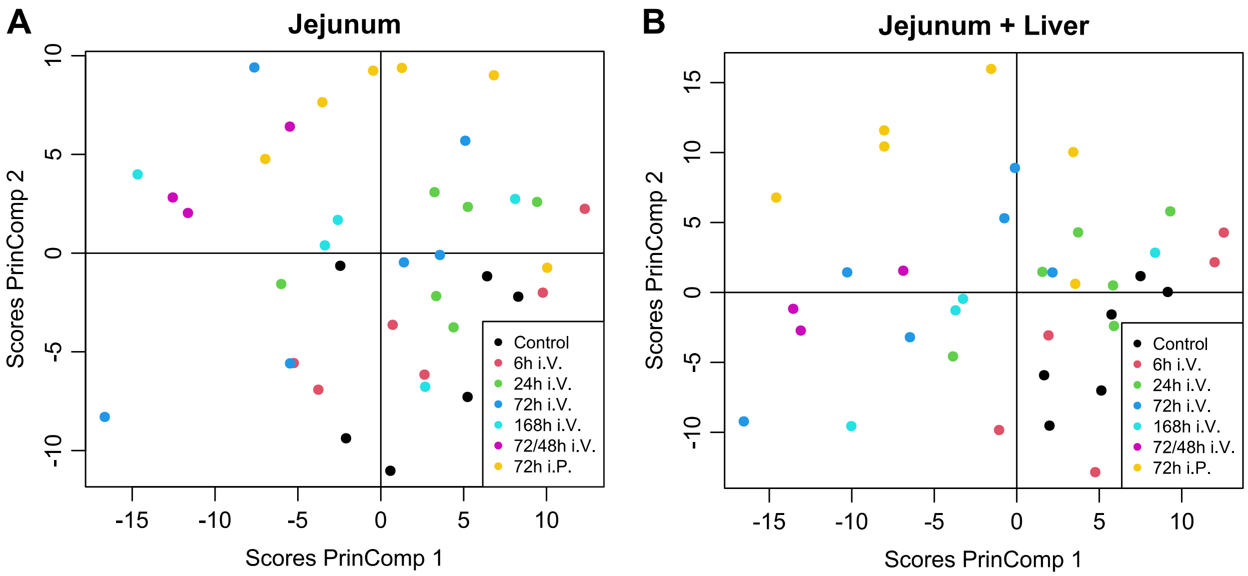

Figure S1. Scatterplot of the scores of the principal component analyses in Figure 2. **A)** Scores of the first two principal components of the lipids identified in the jejunum (the loadings are in Figure 2A). Principal Component 1 represents 32% of the variance and Principal Component 2 represents 19% of the variation. **B)** Scores of the first two principal component analysis of the lipids indentified in the jejunum and the liver (the loadings are in Figure 2B). Principal Component 1 represents 21% of the variance and Principal Component 2 represents 15% of the variation. i.V., intra venous treatment; i.P., intra peritoneal treatment.
